# Caffeic acid and chlorogenic acid cytotoxicity, genotoxicity and impact on global DNA methylation in human leukemic cell lines

**DOI:** 10.1590/1678-4685-GMB-2019-0347

**Published:** 2020-07-03

**Authors:** Lívia Cristina Hernandes, Ana Rita Thomazela Machado, Katiuska Tuttis, Diego Luís Ribeiro, Alexandre Ferro Aissa, Paula Pícoli Dévoz, Lusânia Maria Greggi Antunes

**Affiliations:** 1 Universidade de São Paulo - USP, Faculdade de Ciências Farmacêuticas de Ribeirão Preto Ribeirão Preto, SP, Brazil.; 2 Universidade de São Paulo USP, Faculdade de Medicina de Ribeirão Preto, Ribeirão Preto, SP, Brazil.

**Keywords:** Polyphenols, comet assay, micronucleus, neutral red

## Abstract

Dietary phenolic compounds such as caffeic and chlorogenic acid exert an antiproliferative effect and modulate the gene-specific DNA methylation status in human breast tumor cells, but it remains unclear whether they interfere with global DNA methylation in human leukemia cells. We examined whether caffeic and chlorogenic acid (1-250 µM) exert antitumor action in human promyelocytic leukemia cells (HL-60) and human acute T-cell leukemia cells (Jurkat). Caffeic and chlorogenic acid did not reduce cell viability in the two cell lines, as assessed using the neutral red uptake and MTT assays. These phenolic acids (1-100 μM) neither induced DNA damage (comet assay) nor increased the micronuclei frequency (micronucleus assay) in HL-60 and Jurkat cells, indicating that they were not genotoxic or mutagenic. Analysis of global DNA methylation levels using a 5-mC DNA ELISA kit revealed that chlorogenic acid at a non-cytotoxic concentration (100 μM) induced global DNA hypomethylation in Jurkat cells, but not in HL-60 cells, suggesting that it exerts a cell-specific effect. Caffeic acid did not change global DNA methylation. As other phenolic compounds, chlorogenic acid probably modulates DNA methylation by targeting DNA methyltransferases. The hypomethylating action of chlorogenic acid can be beneficial against hematological malignances whose pathogenic processes involve impairment of DNA methylation.

## Introduction

Coffee is one of the most popular beverages worldwide and has a great impact on the human body as a whole (Buldak *et al.*, 2018). The phytochemical compounds present in coffee are able to inhibit oxidative stress and oxidative damage which is related to early process of the transformation of a normal cell into a malignant tumor (Grosso *et al.*, 2017). Coffee has been demonstrated to play a role in cellular defense mechanisms, including regulating DNA repair and apoptosis, as well as having antiproliferative, antiangiogenic, and antimetastatic effects (Grosso *et al.*, 2017). The coffee polyphenols caffeic acid and chlorogenic acid, exert antitumor effect through activation and inhibition of some important pathways of cancer metabolism (Lukitasari *et al.*, 2018). Moreover, epidemiological studies revealed that high consumption of coffee was related to a lower risk of incidence of specific cancers and metabolic, digestive, hepatic, or disorders of neurological conditions (Buldak *et al.*, 2018). The antitumor action of coffee polyphenols has been investigated in tumor cell lines, pre-clinical, and clinical studies (Rajagopal *et al.*, 2018). Chlorogenic acid induces apoptosis and selectively inhibits proliferation of A498 human renal cancer cells but not of HEK293 human embryonic kidney cells (Wang *et al.*, 2019). Caffeic acid inhibits proliferation of hepatocellular carcinoma cells and SK-Mel-28 human melanoma cancer cells by inducing apoptosis (Brautigan *et al.*, 2018), alters gene expression in SK-Mel-28 cells (Pelinson *et al.*, 2019), and attenuates the properties of cancer stem cell-like cells by increasing DNA methylating and inducing the expression of miR-148a (Li *et al.*, 2015). Both caffeic acid and chlorogenic acid suppress DNA methylation in MCF-7 and MDA-MB-231 human breast cancer cells (Lee and Zhu 2006).

However, *in vitro* assays with polyphenols have controversial results. Chlorogenic acid inhibits the induction of oxidative damage in rat pheochromocytoma cells (Yao, *et al.*, 2019), while caffeic acid protects human erythrocyte membranes from oxidizing species (Colina *et al.*, 2019). Furthermore, caffeic acid at 100 μM induces DNA damage in human lymphocytes (assessed by the comet assay); at 500-1500 μM, it increases the micronucleus frequency in rat hepatoma tissue cells (Szeto *et al.*, 2002; Maistro *et al.*, 2011). The IARC Monographs classify caffeic acid in the group 2B as possibly carcinogenic to humans (IARC, 1993).

Epigenetic dysregulation, such as DNA hypo- or hypermethylation, is closely associated with the development and progression of hematological malignancies. Alterations in the cellular epigenome, such as impaired DNA methylation mediated by DNA methyltransferases, play important roles in tumorigenesis of acute myeloid leukemia and acute lymphoblastic leukemia (Dexheimer *et al.*, 2017; Helmer *et al.*, 2019).

A previous study demonstrated that habitual coffee consumption can alter DNA methylation sites in human genome. It revealed alterations in methylation status of CpG sites located near of 11 genes associated with habitual coffee consumption in peripheral blood mononuclear cells (Chuang *et al.*, 2017).

To the best of our knowledge, there are no reports in the literature evaluating the effects of acid caffeic and acid chlorogenic effects on human leukemic cells. Therefore, in the present study, we examined the antitumor potential of caffeic acid and chlorogenic acid by assessing their cytotoxicity, genotoxicity and effects on global DNA methylation towards human promyelocytic leukemia cells (HL-60) and human acute T-cell leukemia cells (Jurkat).

## Material and Methods

### Chemicals

Acridine orange (CAS: 10127-02-3), caffeic acid (CAS: 331-39-5), chlorogenic acid (CAS: 327-97-9), cytochalasin B (CAS: 14930-96-2), dimethyl sulfoxide (DMSO, CAS: 67-68-5), 3-(4,5-dimethyl-2-thiazolyl)-2,5-diphenyl-2H-tetrazolium bromide (MTT, CAS: 298-93-1), ethylenediamine tetraacetic acid (EDTA, CAS: 60-00-4), methyl methanesulfonate (MMS; CAS: 66-27-3), neutral red (CAS: 553-24-2), and trypan blue (CAS: 72-57-1) were obtained from Sigma-Aldrich (St. Louis, MO, USA). Wizard^®^ Genomic DNA Purification Kit was obtained from Promega (Madison, WI, USA). 5-mC DNA ELISA Kit (colorimetric) was obtained from Zymo Research (Irvine, CA, USA). Antibiotic mix (penicillin/streptomycin/neomycin), fetal bovine serum (FBS), RPMI (*Roswell Park Memorial Institute*) 1640 culture medium, and trypsin-EDTA were acquired from Gibco (Carlsbad, CA, USA). Low melting point (LMP) agarose (CAS: 39346-81-1) and normal melting point (NMP) agarose (CAS: 9012-36-6) were purchased from Invitrogen (Carlsbad, CA, USA). GelRed was acquired from Biotium (Hayward, CA, USA). The other reagents used were of analytical grade.

### Cell culture conditions

All *in vitro* cell culture procedures were performed using a Class II and Type 1A laminar flow cabinet from Bioprotector (VecoFlow Ltda, Campinas, SP, Brazil). Human promyelocytic leukemia cells (HL-60) and human acute T-cell leukemia cells (Jurkat) were obtained from American Type Culture Collection (ATCC, Manassas, VA, USA) and cultured according to the procedures reported by Bal-Price and Coecke (2011). Cells were maintained in RPMI 1640 medium supplemented with 10% FBS, 1% antibiotic-antimycotic solution (5 mg/mL penicillin, 5 mg/mL streptomycin, and 10 mg/mL neomycin), and 0.024% (w/v) NaHCO_3_, in a CO_2_ incubator with 5% atmosphere, at 37 °C, and 95% relative humidity. The medium was changed every 2–3 days. All the experiments were conducted between the third and the eighth cell passage.

### Determination of cell viability

Cell viability was assessed using the neutral red uptake assay described by Repetto *et al.* (2008) and the MTT assay reported by Mosmann (1983). First, HL-60 or Jurkat cells (5 10^4^/mL) were seeded in 96-well plates, in complete culture medium, for 24 h. After stabilization, the cells were treated with caffeic acid or chlorogenic acid (1 – 250 μM), PBS pH 7.4 (negative control), DMSO (0.4% v/v; solvent control) or MMS (100 μM; positive control) for 24 h. Cell viability was determined using the neutral red uptake assay (Repetto *et al.*, 2008). Absorbance of the samples was analyzed using an iMark™ Microplate Absorbance Reader (Agilent Technologies, Santa Clara, CA, USA), set at λ = 540 nm.

Second, cells (1 × 10^4^/well) were seeded in 96-well culture plates and incubated for 24 h before treatment with three concentrations (1, 10, and 100 μM) of caffeic acid or chlorogenic acid, 100 μM MMS (positive control), DMSO (0.4% v/v; solvent control) or PBS pH 7.4 (negative control) for further 24 h. The reaction medium was later removed, a MTT solution (working solution: 10:100 μL v/v; 0.5 mg/mL) was added to the wells, and the plate was incubated for 4 h. Finally, the supernatant was replaced by DMSO, and absorbance was recorded in a spectrophotometer (Biotek Elx800; Winooski, VT, USA) set at λ = 570 nm. The percentage of viable cells was calculated after normalization with the negative control, which was considered as 100% cell viability.

### Comet assay

HL-60 or Jurkat cells (1 × 10^5^/mL) were seeded in 24-well plates, in complete culture medium, for 24 h. After stabilization, the cells were treated with caffeic acid or chlorogenic acid (1 – 100 μM), PBS pH 7.4 (negative control), DMSO (0.4% v/v; solvent control) or MMS (100 μM; positive control), for 4 h. Cell viability was determined by the trypan blue exclusion technique, using a Countess^®^ Automated Cell Counter (Life Technologies, Carlsbad, USA). Cultures with cell viability > 80% were subjected to the comet assay, according to the protocol reported by Tice *et al.* (2000). Finally, the slides were stained with GelRed™ solution and analyzed in a fluorescence microscope (Carl Zeiss, Axiostar Plus, Jena, Germany) using an 515–560 nm excitation filter and a 590 nm filter barrier, with the aid of the Comet Assay IV™ software (Perceptive Instruments, Haverhil, UK).

### Micronucleus assay

HL-60 or Jurkat cells (1 × 10^5^/mL) were seeded in 6-well plates, in complete culture medium, and treated with caffeic acid or chlorogenic acid (1, 10 or 100 μM), PBS pH 7.4 (negative control), DMSO (0.4% v/v; solvent control) or 5-azacytidine (5 μM; positive control), for 24 h. Next, cytochalasin B (final concentration of 6.0 mg/mL) was added to the wells. Immediately before microscopy analysis (Carl Zeiss, Jena, Germany), cells were stained with acridine orange (0.1%; diluted 1:15 v/v in PBS). Binucleated cells with micronuclei (MNi) were scored at 1000 x magnification according to Fenech (2000). A total of 6,000 binucleated cells were analyzed for the presence of MNi. The fold-change in MNi frequency was expressed as the ratio between the mean value of each treatment and the mean value of the negative control (Fenech *et al.*, 2016).

### DNA isolation and global methylation assays

Genomic DNA was extracted from HL-60 or Jurkat cells (5 × 10^4^/mL) treated with caffeic acid or chlorogenic acid (100 μM), PBS pH 7.4 (negative control), or DMSO (0.4% v/v; solvent control), for 72 h. DNA was extracted using the Wizard^®^ Genomic DNA Purification Kit (Promega, Madison, WI, USA) and quantified using a NanoDrop spectrophotometer (Thermo Fisher Scientific, Waltham, MA, USA). Global DNA methylation was analyzed using the 5-mC DNA ELISA Kit (colorimetric) (Zymo Research, Irvine, CA, USA), according to the manufacturers' instructions, and absorbance was recorded at 405 nm (Biotek, Elx800 – Winooski, VT, USA). Global DNA methylation was expressed as % 5-mC.

### Statistical analysis

Statistical analysis was performed using GraphPad Prism 5.0 software (La Jolla, CA, USA). All experiments were carried out in triplicate and the results were expressed as the mean ± standard deviation (SD). The positive and negative control groups were compared using the Student's *t*-test, while the test groups were compared with the negative control group using one-way analysis of variance (ANOVA) combined with the Dunnett's post-hoc test. p<0.05 was considered statistically significant.

## Results

### Caffeic acid and chlorogenic acid are not cytotoxic towards HL-60 and Jurkat cells

**Figure 1:**
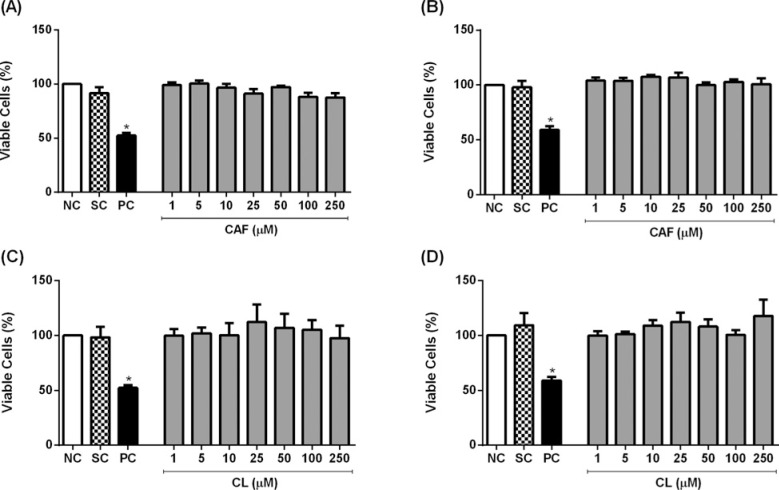
Cell viability of HL-60 (A, C) and Jurkat (B, D) cells treated with caffeic acid (CAF) and chlorogenic acid (CL) for 24 hours, as assessed using the neutral red uptake assay. Negative control (NC) = PBS; Solvent control (SC) = 0.4% DMSO; Positive control (PC) = 100 μM methyl methanesulfonate. Results are expressed as mean ± SD of three independent experiments. *p<0.05 *vs.* negative control (Student's *t* test).

**Figure 2:**
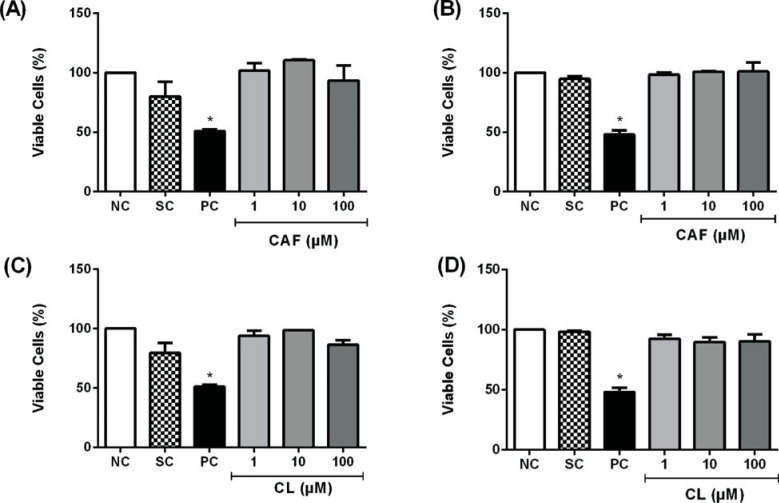
Cell viability of HL-60 (A, C) and Jurkat cells (B, D) treated with caffeic acid (CAF) and chlorogenic acid (CL) for 24 hours, as assessed using the MTT assay. Negative control (NC) = PBS; Solvent control (SC) = 0.4% DMSO; Positive control (PC) = 100 μM methyl methanesulfonate. Results are expressed as mean ± SD of three independent experiments. *p <0.05 *vs.* negative control (Student's *t* test).

We examined whether caffeic acid and chlorogenic acid affected the cell viability of HL-60 and Jurkat cells, using the neutral red uptake and MTT assays. MMS, but not the phenolic acids at concentrations up to 250 μM, reduced cell viability assessed by the neutral red uptake assay (Figure 1). Similar results were obtained when the cells were treated with the phenolic acids at concentrations up to 100 μM, and cell viability was assessed using the MTT assay (Figure 2). Hence, caffeic acid and chlorogenic acid were not cytotoxic to HL-60 and Jurkat cells.

#### Caffeic acid and chlorogenic acid are not genotoxic to HL-60 and Jurkat cells

**Figure 3:**
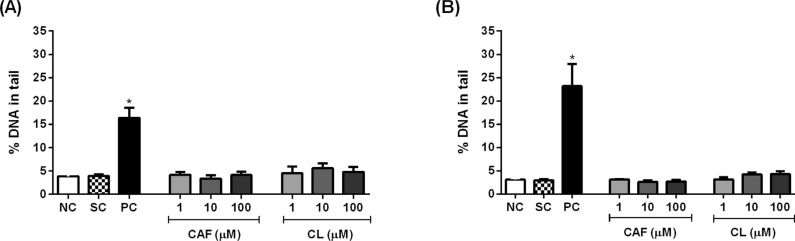
Percentage of DNA in the tail in HL-60 (A) and Jurkat (B) cells treated with caffeic acid (CAF) and chlorogenic acid (CL) for 4 hours. Negative control (NC) = PBS; Solvent control (SC) = 0.4% DMSO; Positive control (PC) = 100 μM methyl methanesulfonate. Results are expressed as mean ± SD (n = 3). *p <0.05 *vs.* negative control (Student's *t* test).

We used the comet assay to examine whether caffeic acid and chlorogenic acid induced DNA damage in HL-60 and Jurkat cells. MMS, but not the phenolic acids at concentrations up to 100 μM, increased the percentage of DNA in the tail in both cell lines (Figure 3). MMS induced a stronger DNA damage in Jurkat cells than in HL-60 cells, as demonstrated by the increased percentage of DNA in the tail.

**Figure 4:**
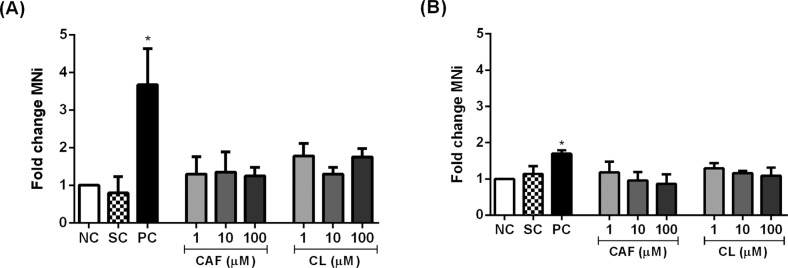
Fold change in micronuclei (MNi) frequency per 1,000 binucleated HL-60 (A) and Jurkat (B) cells treated with caffeic acid (CAF) and chlorogenic acid (CL). Negative control (NC) = PBS; Solvent control (SC) = 0.4% DMSO; Positive control (PC) = 5 μM 5-azacytidine. Results are expressed as mean ± SD (n = 3). *p <0.05 *vs.* negative control (Student's *t* test).

We expressed the MNi frequency as fold change relative to the negative control group because the MNi frequency in this group differed between HL-60 and Jurkat cells. The fold change in MNi frequency in both cell lines treated with caffeic acid and chlorogenic acid was similar to negative control (p > 0.05) (Figure 4).

#### Chlorogenic acid decreases global DNA methylation in Jurkat cells

**Figure 5:**
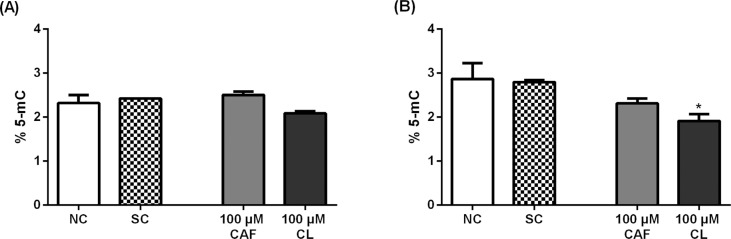
Global DNA methylation (% 5-mc) in HL-60 (A) and Jurkat (B) cells treated with caffeic acid (CAF) and chlorogenic acid (CL) for 72 hours. Negative control (NC) = PBS; Solvent control (SC) = 0.4% DMSO. Results are expressed as mean ± SD (n = 3). *p <0.05 *vs.* negative control (One-way ANOVA followed by the Dunnett's post-hoc test).

Compared with the negative control, the 72-h treatment with 100 μM chlorogenic acid, but not with caffeic acid, significantly decreased the levels of % 5-mC in Jurkat cells (p<0.05); in HL-60 cells, both phenolic acids did not alter the levels of % 5-mC (Figure 5).

## Discussion

No reports of toxicity were found in humans exposed to caffeic and chlorogenic acids. However, in a study performed with rats, the intraperitoneal dose of 11.29 mmol/kg of chlorogenic acid induced death in 4 of 6 treated animals, while doses less than 6.878 mmol/kg were nontoxic. The dose of 8.326 mmol/kg of caffeic acid induced death in 5 of 8 treated rats, but doses lower than 6.938 mmol/kg were nontoxic (Chaube and Swinyard, 1976).

In humans, the absorption was measured after the ingestion of 2.8 mmol of each acid, resulting in 33% of chlorogenic acid and 95% of caffeic acid. This study showed that about one third of chlorogenic acid and almost 100% of the caffeic acid were absorbed in the small bowel of humans (Olthof *et al.*, 2001). Therefore, concentrations of 1 - 250 µM of caffeic and chlorogenic acids were chosen to be used in the present study.

In this study, we addressed whether caffeic acid and chlorogenic acid induced cytotoxicity, genotoxicity, and modify DNA global methylation in HL-60 and Jurkat cells. Both phenolic acids were not cytotoxic to the two cell lines, when tested at 1–250 μM and 1–100 μM using the neutral red uptake and MTT assays, respectively.

Caffeic acid suppresses proliferation of hepatocellular carcinoma cells by inducing apoptosis (Brautigan *et al.*, 2018), inhibits migration of MCF-7 breast cancer cells (at 50-100 μM) (Kabala-Dzik *et al.*, 2018), and suppresses proliferation and migration of A549 human lung cancer cells and HT29-D4 colon adenocarcinoma cells (Nasr Bouzaiene *et al.*, 2015). Chlorogenic acid decreases viability of HCT116 and HT29 human colon cancer cells (Hou *et al.*, 2017) and induces apoptosis in A2780 and A2780CP70 human ovarian cancer cells *in vitro* (Tai *et al.*, 2010), but it has no significant effects on proliferation of HepG2 human hepatoma cells (Ramos *et al.*, 2005).

Our findings corroborate a recent study that investigated the antiproliferative effect of six coffee compounds, including kahweol acetate, cafestol, caffeine, caffeic acid, chlorogenic acid, and trigonelline hydrochloride on LNCaP, LNCaP-SF, PC-3, and DU145 human prostate cancer cells (Iwamoto *et al.*, 2019). Chlorogenic acid and caffeic acid at concentrations ranging from 5 to 50 µM do not inhibit proliferation and migration of human prostate cancer cells (Iwamoto *et al.*, 2019). Caffeic acid is also not cytotoxic towards MDA-MB-231 cells, a triple-negative human caucasian breast adenocarcinoma cell line, affording IC_50_ values greater than 10,000 and 1,000 µM after 24 and 48 h of treatment, respectively (Kabala-Dzik *et al.*, 2017).

Caffeic acid and chlorogenic acid (1 – 100 μM) were not genotoxic or mutagenic to HL-60 or Jurkat cells, since these phenolic acids did not induce DNA damage (assessed by the comet assay) or increased MNi frequencies (assessed by the micronucleus test). Caffeic acid does not cause DNA damage in human peripheral blood mononuclear cells and HL-60 cells (Fabiani *et al.*, 2008), but induces DNA damage in human lymphocytes at concentrations as high as 1500 μM (Maistro *et al.*, 2011), as assessed using the comet assay. This phenolic acid also reduces DNA damage in UVBirradiated lymphocytes, increases MNi frequency in Hep3B cells, but it does not alter MNi frequency in HepG2 cells, at concentrations ranging from 10 to 1000 μM (Majer *et al.*, 2004; Prasad *et al.*, 2009). Chlorogenic acid (20 µM) protects HaCaT human keratinocytes from UVB-induced oxidative damage (Cha *et al.*, 2014).

The controversial cytotoxic and genotoxic effects of these phenolic acids may be related to the presence of transition metal ions and their pro-oxidant action (Bhat *et al.*, 2007). In the present study, HL-60 and Jurkat cells were cultured in RPMI 1640 medium, which contains the reducing agent glutathione and high concentrations of vitamins. Recently, Grzesik *et al.* (2019) reported H_2_O_2_ generation associated with oxidation of phenolic compounds, including caffeic acid and chlorogenic acid, in Dulbecco's modified Eagle's medium (DMEM). DMEM contains iron while RPMI 1640 contains only trace concentrations of iron, suggesting that this transition metal promotes auto-oxidation of these compounds.

Downregulation of DNA methylation is an important molecular event that modulates the pathogenesis of hematological malignancies. Hypomethylating agents have been used as therapeutic alternatives to treat myelodysplastic syndromes and acute myeloid leukemia in elderly patients (Santini *et al.*, 2013; Yun *et al.*, 2016). Analysis of global DNA methylation revealed that non-treated HL-60 and Jurkat cells exhibited 2.3% and 2.9% of 5-mC DNA, respectively. The 5-mC levels in primary human fibroblasts ranged from 1.0% to 1.7% (Maierhofer *et al.*, 2017), while the mean 5-mC level was 2.8% in peripheral blood of 100 male workers employed in automotive battery factories (Devoz *et al.*, 2017), 3.82% in leukocytes from 390 volunteers with no known cancer (Shen *et al.*, 2017), and 2.28% in non-treated LNCaP human prostate cancer cells *in vitro* (Wu *et al.*, 2017).

A three-day treatment with caffeic acid (1 – 50 mM) and chlorogenic acid (1 – 20 mM) decreases the methylation-specific band for the retinoic acid receptor beta (*RARB*) but does not significantly change global DNA methylation in MCF-7 and MDA-MB-231 human breast cancer cells (Lee and Zhu, 2006). Tumor cells with elevated levels of DNA methyltransferases can be sensitive to treatment with hypomethylating agents, while cells deficient in these enzymes can be resistant to these agents (Juttermann *et al.*, 1994).

In the present study, chlorogenic acid at a non-cytotoxic concentration (100 μM) induced global DNA hypomethylation in Jurkat cells but not in HL-60 cells, suggesting that this phenolic acid exerts a cell-specific action. The possible mechanism by which chlorogenic acid modulates DNA methylation is by targeting DNA methyltransferases (DNMTs), in a manner similar to that described for polyphenols (Li *et al.*, 2016; Nasir *et al.*, 2020).

Polyphenols compounds have a catechol group in their constitution, a key substrate for O-methylation of catecholamines mediated by the enzyme catechol-O-methyltransferase (COMT) (Lee *et al.*, 2005; Kadayifci, *et al.*, 2018). COMT increases the formation of S-adenosylhomocysteine (SAH), a well-known non-competitive inhibitor of DNMTs (Oliva *et al.*, 1980; Lee *et al.*, 2005). Global DNA methylation is decreased after DNMT1 inhibition (Christman, 2002). In fact, Lee *et al.* (2005) showed that caffeic and chlorogenic acids decreased the DNA methylation catalyzed by human DNMT1. Our results showed that chlorogenic acid decreased DNA methylation in Jurkat cells but not in HeLa cells. This suggests that the mechanisms by which chlorogenic acid acts could be cell-type specific and highlights the importance of using more than one cell line when studying the effects of compounds on DNA methylation.

The nucleoside analogues 5-azacytidine and 5-aza-2-deoxycytidine have been used as hypomethylating agents for over a decade in the chemotherapeutic treatment of acute myeloid leukemia (Leung *et al.*, 2019). Considering the strong cytotoxicity of nucleoside analogues, additional studies are needed to develop innovative therapeutic strategies based on the use of non-nucleoside analogues. In this sense, plant-derived compounds are promising inhibitors of DNA methyltransferases (Schneider-Stock *et al.*, 2012).

In conclusion, caffeic acid and chlorogenic acid are not cytotoxic or genotoxic towards HL-60 and Jurkat cells. Chlorogenic acid induces cell-specific hypomethylation in Jurkat cells, which can be beneficial against hematological malignances where impaired DNA methylation plays an important role in the pathogenic process.
